# Effect of straw return on soil respiration and *NEE* of paddy fields under water-saving irrigation

**DOI:** 10.1371/journal.pone.0204597

**Published:** 2018-10-16

**Authors:** Shihong Yang, Yanan Xiao, Junzeng Xu, Xiaoyin Liu

**Affiliations:** 1 State Key Laboratory of Hydrology-Water Resources and Hydraulic Engineering, Hohai University, Nanjing, P.R. China; 2 College of Agricultural Engineering, Hohai University, Nanjing, P.R. China; Tennessee State University, UNITED STATES

## Abstract

Straw return (SR) and rice water-saving irrigation (WSI) affect the greenhouse gas emission of paddy fields. However, studies on CO_2_ exchange between paddy fields and the atmosphere with joint regulation of SR and WSI are few. We conducted a two-year field experiment to investigate the effects of SR on soil respiration and net ecosystem exchange of CO_2_ (*NEE*) in paddy fields under controlled irrigation (CI), which is a typical WSI technique. The rice yields, irrigation water use efficiency, seasonal variations in soil respiration, *NEE*, and soil organic carbon content were measured. Compared with the control (flooding irrigation and traditional chemical fertilizer), a significant increase in rice yield and irrigation water use efficiency in the paddy fields under CI and SR joint management (CS) was observed. CS increased the soil respiration rate during most of the rice growth stage and increased the net CO_2_ absorption rate before approximately 80 days after transplanting; afterward, the pattern reversed. Total CO_2_ emissions through soil respiration in CS paddy fields increased by 43.7% and 182% compared with the control in 2014 and 2015, respectively. However, CS also caused an increase in the total net CO_2_ absorption by 18.1% and 30.1% in these two years, respectively. The acceleration in the consumption and decomposition of soil organic carbon induced by frequent alternate wet–dry cycles of the CI paddy fields increased the soil respiration and decreased the net CO_2_ absorption. SR promoted soil respiration but also improved rice growth, increasing the net CO_2_ absorption. The soil organic carbon content of the CS paddy fields after harvesting increased by 23.2% compared with that before transplanting. The present study concluded that joint regulation of WSI and SR is an effective measure for maintaining yield, increasing irrigation water use efficiency, mitigating CO_2_ emission, and promoting paddy soil fertility.

## Introduction

The increasing use of straw return (SR), an important management practice in global organic agriculture [1], is recommended to decrease chemical inputs, promote soil C sequestration, and improve crop yields [2–5]. However, SR has been shown to increase greenhouse gas (GHG) emissions. For example, Zhang et al. [6] showed that SR increased the soil respiration rate by 9.60% in dry farmland. Liu et al. [7] found that SR increased methane (CH_4_) emissions by 111% in rice paddies and increased nitrous oxide (N_2_O) emissions by 90.0% in upland soils. Agricultural ecosystems are a major source of GHG emissions. The average annual total GHG emissions from agriculture reached 5.00–5.80 Gt CO_2_ eq year^−1^ in 2000–2010, accounting for approximately 12.0% of total anthropogenic GHG emissions [8]. Thus, the environmental effects of SR application require comprehensive evaluation.

Rice is one of the most important cereal crops in the Asian monsoon region, and in 2014, the harvest area in this region was 143 million ha, accounting for 88% of the total rice harvest area worldwide [9]. With increasing water scarcity due to climate change, water-saving irrigation (WSI) techniques are being widely implemented in rice paddies [10,11]. The common point of these WSI techniques is non-flooding management or in unsaturated state of paddy soil during some or most of the rice growth season, leading to water conditions that are different from traditional flooding irrigation (FI). Thus, under such management, paddy fields experience multiple dry–wet cycles and consequently undergo changes in soil biological and chemical processes [12]. These results lead to changes that improve the effects of SR on GHG emissions from paddy fields. Zou et al. [13] found that under a water regime of flooding midseason, drainage, reflooding, moist intermittent (F-D-F-M) irrigation, wheat straw and rapeseed cake incorporation resulted in a 252% increase in CH_4_ emissions; moreover, rapeseed cake increased N_2_O by 17.0%, and wheat straw reduced N_2_O by 19.0% compared with controls. However, double rice-cropping system experiments indicated that midseason drainage and F-D-F-M irrigation reduced CH_4_ emissions by 52.5% and 69.3% from paddy fields with SR, respectively, whereas F-D-F-M increased N_2_O emissions by 60.9% [14]. Existing studies on the effects of SR on GHG emission from paddy fields under WSI have focused mainly on CH_4_ and N_2_O emissions [15–18].

Carbon dioxide (CO_2_) is another important GHG emitted from farmlands. When paddy soil is exposed to multiple wet–dry cycles under WSI, emission patterns and total emissions of CO_2_ under SR will change. However, limited information on this change is available. The soil respiration and net ecosystem exchange of CO_2_ (*NEE*) are two important characteristic values of CO_2_ exchange between farmland and the atmosphere. Using the widely adopted WSI technique, we conucted a field experiment to identify the influence of SR on soil respiration and *NEE* of paddy fields under non-flooding controlled irrigation (CI) management. The objectives of this study were to (1) reveal the effects of water and carbon management on rice yield and irrigation water use efficiency; (2) analyze and compare the characteristics of seasonal variation in the soil respiration rate and the *NEE* of a paddy field ecosystem under different types of water and carbon management; (3) quantify total CO_2_ emissions through soil respiration (total *R*_*soil*_) and the total *NEE* of a paddy field ecosystem; and (4) discuss the effects of water and carbon management on soil respiration and the *NEE* of a paddy field ecosystem. The results can support more comprehensive evaluations of the ecological and environmental effects of rice WSI and SR. At the same time, they will also contribute to the comprehensive evaluation of GHG emissions and the sustainable use of water and carbon resources of paddy fields in China.

## Materials and methods

### Site description

The field experiment was conducted in 2014–2015 at the Kunshan Irrigation and Drainage Experiment Station in the Taihu Lake Region of China (31°15′15″ N latitude, 120°57′43″ E longitude). A rice–wheat rotation is used in this region. The paddy soil in the experimental site is a clay-textured hydragric anthrosol (75.0% clay, 16.2% silt, and 8.80% sand) with 21.9 g kg^−1^ organic matter, 1.03 g kg^−1^ total nitrogen, 1.35 g kg^−1^ total phosphorus, 20.9 g kg^−1^ total potassium, and a pH of 7.40. In 2014 and 2015, the mean temperatures were 24.5°C and 24.4°C, and precipitations were 443 and 450 mm during the experimental period, respectively.

### Field management

The experiment was laid out (plot size 150 m^2^) in a randomized block design with four treatments and three replicates. The four treatments were a combination of irrigation and fertilizer managements: the two irrigation managements were CI and FI, and the two fertilizer managements were farmers’ fertilization practice (FFP) and wheat SR. Then, the four treatments were FF (FI and FFP), CF (CI and FFP), FS (FI and SR), and CS (CI and SR). All treatments were applied to the same plots for both years of the study. Rain-fed wheat was grown in the plots during the non-rice season. Non-flooding management was carried out in the CI treatment except for the shallow flooding water during the regreening stage. Moreover, irrigation was applied to saturate the soil only when the soil moisture approached the low threshold in a certain stage, as listed in Table 1. For the FI treatment, 3–5 cm of standing water was maintained in the paddy field except during mid-drainage in the late tillering stage. The soil moisture and water table were monitored at 8:00 a.m. every day throughout the rice growth stage.

**Table 1:** Controlled thresholds in different stages for controlled irrigation.

Limit	Regreening stage	Tillering stage			Jointing and booting stage	Heading and flowering stage	Milk stage	Ripening stage
		Initial	Middle	Late				
Upper limit ^2^	25 mm^1^	100%*θ*_s1_	100%*θ*_s1_	100%*θ*_s1_	100%*θ*_s2_	100%*θ*_s3_	100%*θ*_s3_	Naturally drying
Lower limit	5 mm^1^	70%*θ*_s1_	65%*θ*_s1_	60%*θ*_s1_	75%*θ*_s2_	80%*θ*_s3_	70%*θ*_s3_	
Observed root zone depth (cm)	—	0–20	0–20	0–20	0–30	0–40	0–40	

^1^ Data show the water depth during the regreening stage. θ_s1_, θ_s2_, and θ_s3_ represent average volumetric soil moisture for the 0–20, 0–30, and 0–40 cm layers, respectively.

^2^ In the case of pesticide, fertilizer applications and rainfall, standing irrigation water at a depth of up to 5 cm is maintained for less than five days.

Before the application of fertilizers, herbicides, and pesticides, all the plots were flooded to a depth of 3–5 cm. In addition, the same kinds and amounts of herbicides and pesticides were applied in both irrigation managements.

The rice variety used in this experiment was Japonica Rice Nanjing 46. Three to four seedlings per hill were transplanted with 13.0 cm × 25.0 cm hill spacing in late June and harvested in late October. Local nitrogen fertilizer was adopted in this experiment, as shown in Table 2. In addition to nitrogen fertilizer input, the same phosphorus and potassium fertilizers were applied to all treatments (56.3 kg P_2_O_5_ ha^−1^ and 56.3 kg K_2_O ha^−1^ in 2014, 54.0 kg P_2_O_5_ ha^−1^ and 76.5 kg K_2_O ha^−1^ in 2015). The chemical fertilizer management of the SR treatment was similar to that of the FFP treatment, and 3000 kg ha^−1^ of straw from the previous wheat crop (the organic carbon content of wheat straw was 441 g kg^−1^, and the organic carbon input through wheat straw was 1322 kg ha^−1^) was returned to the SR paddy fields in both years. Base fertilizer and wheat straw were incorporated into the soil during tillage, and surface application was adopted for all other fertilizers.

**Table 2:** Date and rate of nitrogen fertilization during the rice-growing season (kg N ha^-1^).

Activty	2014	2015
Base fertilizer (19 and 29 Jun)	159(56.3CF+103AB)	155(72.0CF+83.2AB)
Tillering fertilizer (29 Jun and 5 Jul)	76.2(U)	69.3(U)
Panicle fertilizer (10 and 9 Aug)	55.4(U)	58.9(U)
Total nitrogen	291	283

Date in the bracket is the time for the fertilizer applied in 2014 and 2015 respectively.

CF: compound fertilizer (N, P_2_O_5_ and K_2_O contents are 15.0%, 15.0% and 15.0% in 2014, and 16.0%, 12.0% and 17.0% in 2015), AB: ammonium bicarbonate (N content is 17.1%), U: urea (N content is 46.2%).

### Field measurement and sampling

Soil respiration and *NEE* of the paddy fields were measured using a transparent static chamber WEST Systems portable soil flux meter (West Systems S.r.l., Italy), which was described in detail previously [19]. During the rice growth stage, soil respiration and *NEE* were measured in every plot at 10:00–11:00 a.m. at 7-day intervals. Sampling bases with and without rice were used to measure the *NEE* and soil respiration fluxes of paddy fields, respectively. The cumulative CO_2_ emissions through soil respiration (total *R*_soil_) and *NEE* of the paddy field ecosystem during the study period were estimated by integrating emission fluxes across time.

Time domain reflectometer (Soil Moisture Equipment, Ltd., Corp. USA) and vertical rulers were used to monitor soil moisture and water depths, respectively. A water meter (Xiamen Longteng Industrial Co., Ltd., China) was installed on the pipe for each plot to measure the irrigation water volumes. The yield for each plot was measured after the rice ripened. Irrigation water use efficiency was calculated by dividing the yield by the irrigation water volume. The soil samples were collected before transplanting and after harvesting in three replicates in each plot at 0–10, 10–20, and 20–40 cm depths. The soil samples for given depths were mixed and analyzed in the laboratory. Soil organic carbon content was determined using the potassium dichromate external heating method [20].

Statistical analyses were performed using standard procedures for a randomized plot design (SPSS 13.0, SPSS Inc., Chicago, IL). Significance was calculated using F-tests, and least significant differences were measured at the 0.05 probability level.

## Results

### Rice yield and irrigation water use efficiency

**Fig 1:**
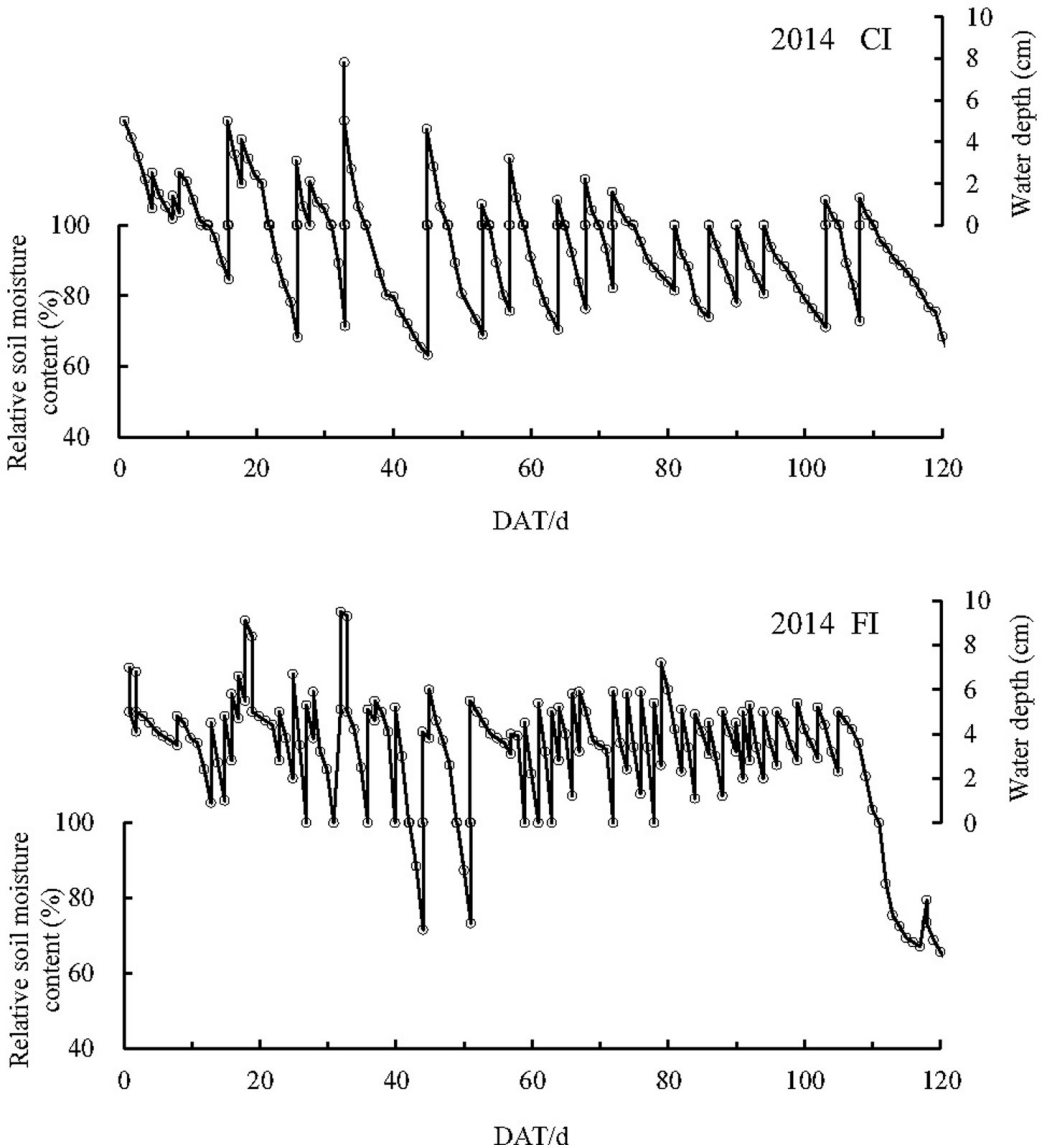
Typical water depth and soil moisture conditions in CI and FI paddy fields in 2014 CI: Controlled irrigation, FI: Flooding irrigation, DAT: Day after transplantation.

**Fig 2:**
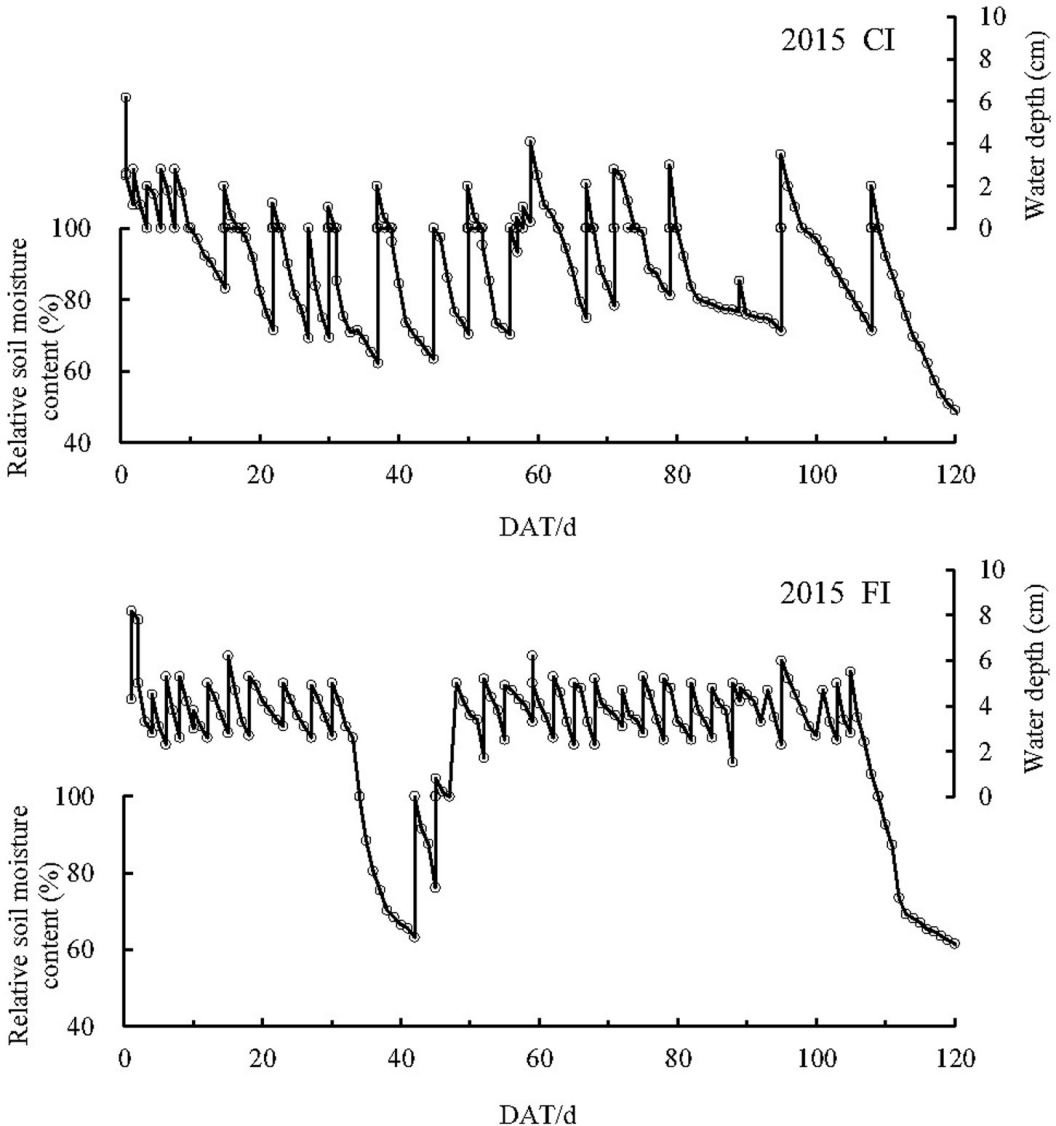
Typical water depth and soil moisture conditions in CI and FI paddy fields in 2015 CI: Controlled irrigation, FI: Flooding irrigation, DAT: Day after transplantation.

Fifteen and thirteen wet–dry cycles occurred in the CI paddy fields in 2014 and 2015, respectively, representing more than 80 days of non-flooding conditions in both years (Figs 1 and 2). For the FI treatments, flooding was maintained, except during 42–44 and 49–51 days after transplantation (DAT) in 2014 and 34–45 DAT in 2015. These periods corresponded to the drainage in the late tillering stage to restrain nonproductive tillering.

No significant difference in rice yield was observed between the different irrigation treatments (Table 3). However, water input was dramatically reduced in the CI treatment by 49.5% and 43.3% in 2014 and 2015 (*p*<0.05), respectively, compared with FI paddy fields. Given the significant reduction in irrigation water input, CI treatment obviously improved the irrigation water use efficiency of the paddy fields. Irrigation water use efficiencies of the CI paddy fields with different carbon managements were increased by 96.4% and 75.8% compared with those of the FI fields in 2014 and 2015 (*p*<0.05), respectively.

**Table 3:** Rice yield and irrigation water use efficiency.

Items		FF	FS	CF	CS
2014	Yield (kg ha^-1^)	9657±54.4b	10422±132a	9589±88.2b	10347±70.8a
	Irrigation water volume (mm)	804±11.0a	804±11.0a	407±5.50b	407±5.50b
	IWUE (kg m^-3^)	1.20±0.0104d	1.30±0.00702c	2.36±0.0241b	2.55±0.0190a
2015	Yield (kg ha^-1^)	9730±40.3b	10119±112a	9682±61.8b	10096±80.4a
	Irrigation water volume (mm)	912±11.9a	912±11.9a	517±5.73b	517±5.73b
	IWUE (kg m^-3^)	1.07±0.0964d	1.11±0.00418c	1.87±0.00875b	1.95±0.00695a

FF: flooding irrigation and farmers’ fertilization practice, FS: flooding irrigation and wheat straw return at a rate of 3000 kg ha^-1^, CF: controlled irrigation and farmers’ fertilization practice, CS: controlled irrigation and wheat straw return at a rate of 3000 kg ha^-1^, IWUE: irrigation water use efficiency. Means in the same line in 2014 or 2015 followed by the same letter are not significantly different (*p <* 0.05).

SR significantly increased rice yield and irrigation water use efficiencies of paddy fields (*p*<0.05). Rice yield and irrigation water use efficiencies of the SR paddy fields under different irrigation treatments increased by 4.14%–7.91% compared with those of the FFP paddy fields. The interaction effect of irrigation and fertilizer treatments on rice yield was not significant but was significant for irrigation water use efficiency (Table 4). In addition to a significant reduction in irrigation water input, the joint regulation of WSI and SR also increased the rice yields and irrigation water use efficiencies compared with traditional irrigation and fertilizer management. Compared with FF paddy fields, the rice yields of the CS paddy fields increased by 7.14% and 3.77% in 2014 and 2015 (*p*<0.05), respectively. Irrigation water use efficiencies of the CS paddy fields were 2.12 and 1.83 times higher than those of FF paddy fields in 2014 and 2015, respectively.

**Table 4:** MANOVA results for rice yield and irrigation water use efficiency.

Year	Influence factor	Rice yield			Irrigation water use efficiency		
		*SS*	*F*	*P*	*SS*	*F*	*P*
2014	Fertilizer management	1.73×10^6^	69.9	3.18×10^−5^*	5.94×10^−2^	71.7	2.89×10^−5^*
	Water management	1.54×10^4^	0.617	0.455	4.35	5.25×10^3^	1.47×10^−12^*
	Interactive effect	33.2	1.33×10^−3^	0.972	6.30×10^−3^	7.60	2.48×10^−2^*
	Error	1.99×10^5^			6.63×10^−3^		
2015	Fertilizer management	4.84×10^4^	26.4	8.89×10^−4^*	1.13×10^−2^	63.9	4.40×10^−5^*
	Water management	3.70×10^3^	0.202	0.665	2.04	1.16×10^4^	6.23×10^−14^*
	Interactive effect	478	2.60×10^−2^	0.876	1.06×10^−3^	6.02	3.98×10^−2^*
	Error	1.47×10^5^			1.41×10^−3^		

*SS*: sum of squares of mean deviation.

*: significant at 0.05 level

### Seasonal variations in soil respiration

**Fig 3:**
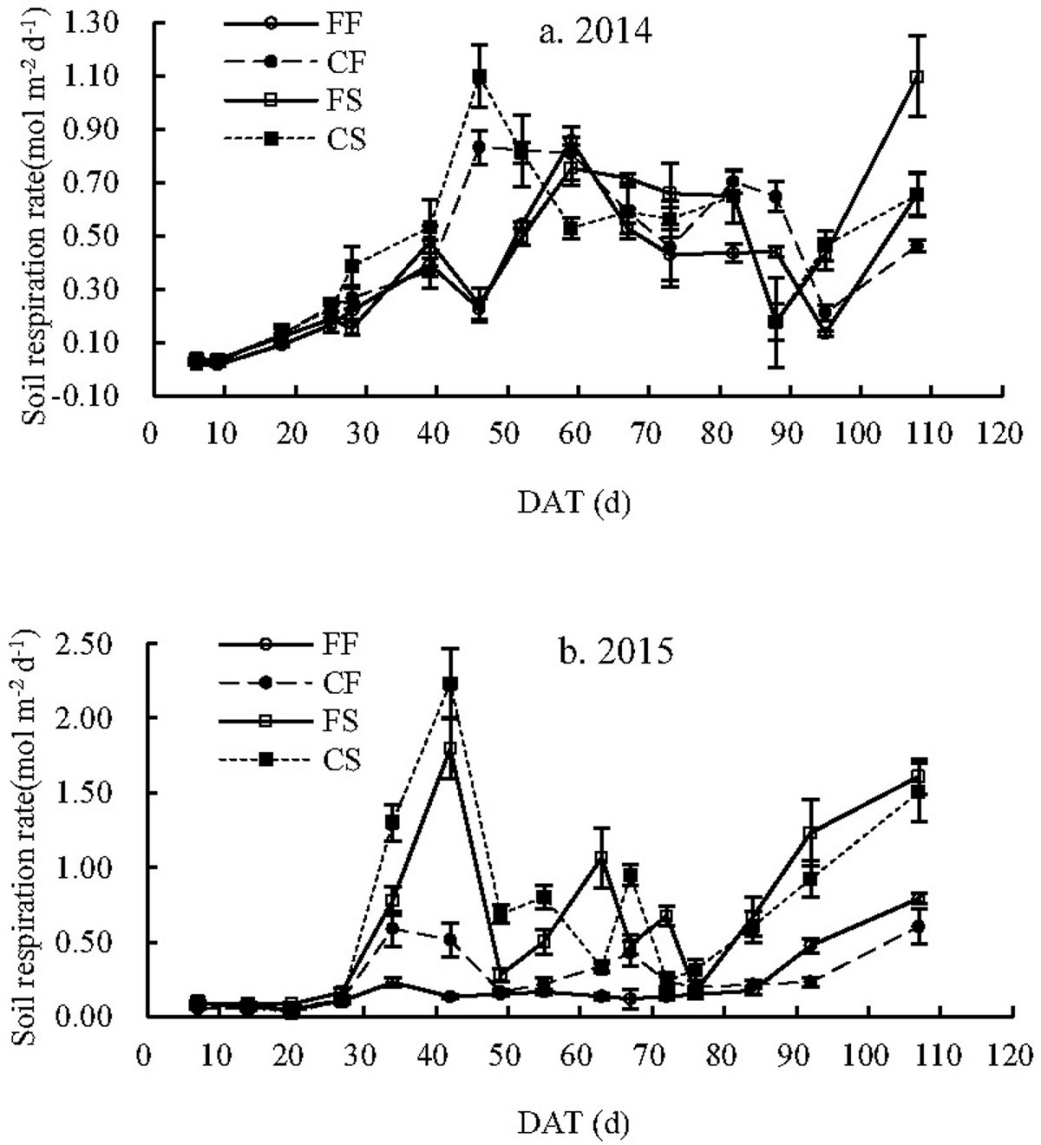
Soil respiration rates of paddy fields with different water and carbon managements FF: Flooding irrigation and farmers’ fertilization practice, FS: Flooding irrigation and wheat straw return at a rate of 3000 kg ha^-1^, CF: Controlled irrigation and farmers’ fertilization practice, CS: Controlled irrigation and wheat straw return at a rate of 3000 kg ha^-1^, DAT: Day after transplantation.

Soil respiration rates exhibited an upward trend after transplanting and peaked during late July or August. Then, they fluctuated but generally exhibited a downward trend until the late milk stage (Fig 3). During the ripening stage, soil respiration rates showed a significant increase during the natural drying period.

The soil respiration enhancement effects of WSI differed between different fertilizer treatments. Before the ripening stage (before 110 DAT), the soil respiration rates of the CI paddy fields with FFP were mostly larger than those of the FI paddy fields. For the SR paddy fields, soil respiration rates of the CI paddy fields were only larger than those of the FI paddy fields before the beginning of late August (approximately 55 DAT). The values of soil respiration rates under different irrigation treatments also crossed each other before the ripening stage. However, the soil respiration rates of the FI paddy fields were greater than those of the CI paddy fields during the ripening stage regardless of fertilizer treatment. For the FFP paddy fields, the average soil respiration rates of the CI paddy fields were 0.438 and 0.267 mol m^−2^ day^−1^ in 2014 and 2015, representing increases of 27.9% and 35.7% compared with those of the FI paddy fields, respectively. However, for the SR paddy fields, the increase in WSI on soil respiration diminished. In addition, the average soil respiration rates of the CI paddy fields increased by 10.9% and 5.23% compared with those in the FI paddy fields in 2014 and 2015, respectively. This interannual variation in soil respiration under the WSI treatment may be attributed to the following factors. Irrigation treatment was the main factor that influenced soil respiration in 2014 due to the similar soil respiration rates of paddy fields under the same irrigation treatments during the rice growth stage. In addition, the soil respiration peaks of paddy fields under the same irrigation treatments occurred at the same time in both years of the study. However, SR had a greater effect on soil respiration in the second year.

SR increased the soil respiration rates of the paddy fields, but the effect differed across irrigation treatments and years. For the CI paddy fields, soil respiration rates of the SR paddy fields were consistently higher than those of the FFP paddy fields before the middle of August (approximately 50 DAT) in 2014. Then, the values of soil respiration rates with different fertilizer managements converged. In the same year, the soil respiration rates of the FS paddy fields were higher than those of the FF paddy fields across most growth stages. In 2015, the soil respiration rates of the SR paddy fields were significantly higher than those of the FFP paddy fields under different water managements (*p*<0.05). There was an interannual difference in the increase in soil respiration associated with SR. In 2014, the average soil respiration rates of the FS and CS paddy fields were 0.413 and 0.458 mol m^−2^ day^−1^, respectively. Moreover, these values were 20.5% and 4.49% higher than those of the FF and CF paddy fields, respectively. The enhanced effect of SR on soil respiration in the second year was obviously higher than that in the first year. The average soil respiration rates of FS and CS paddy fields were 0.644 and 0.678 mol m^−2^ day^−1^ and were 3.27 and 2.53 times higher in 2014 than those of FF and CF paddy fields in in 2015, respectively. In addition, soil respiration peaks of fields under the SR treatments occurred on the same day. The maximum soil respiration rates of the FS and CS paddy fields both occurred at 42 DAT. Moreover, the peak values were 1.80 and 2.23 mol m^−2^ day^−1^ in 2015, respectively.

Average soil respiration rates of the CS paddy fields were 0.457 and 0.678 mol m^−2^ day^−1^ in 2014 and 2015, showing an increase of 33.6% and 244%, respectively, compared with those of FF paddy fields.

### Seasonal variations in *NEE*

**Fig 4:**
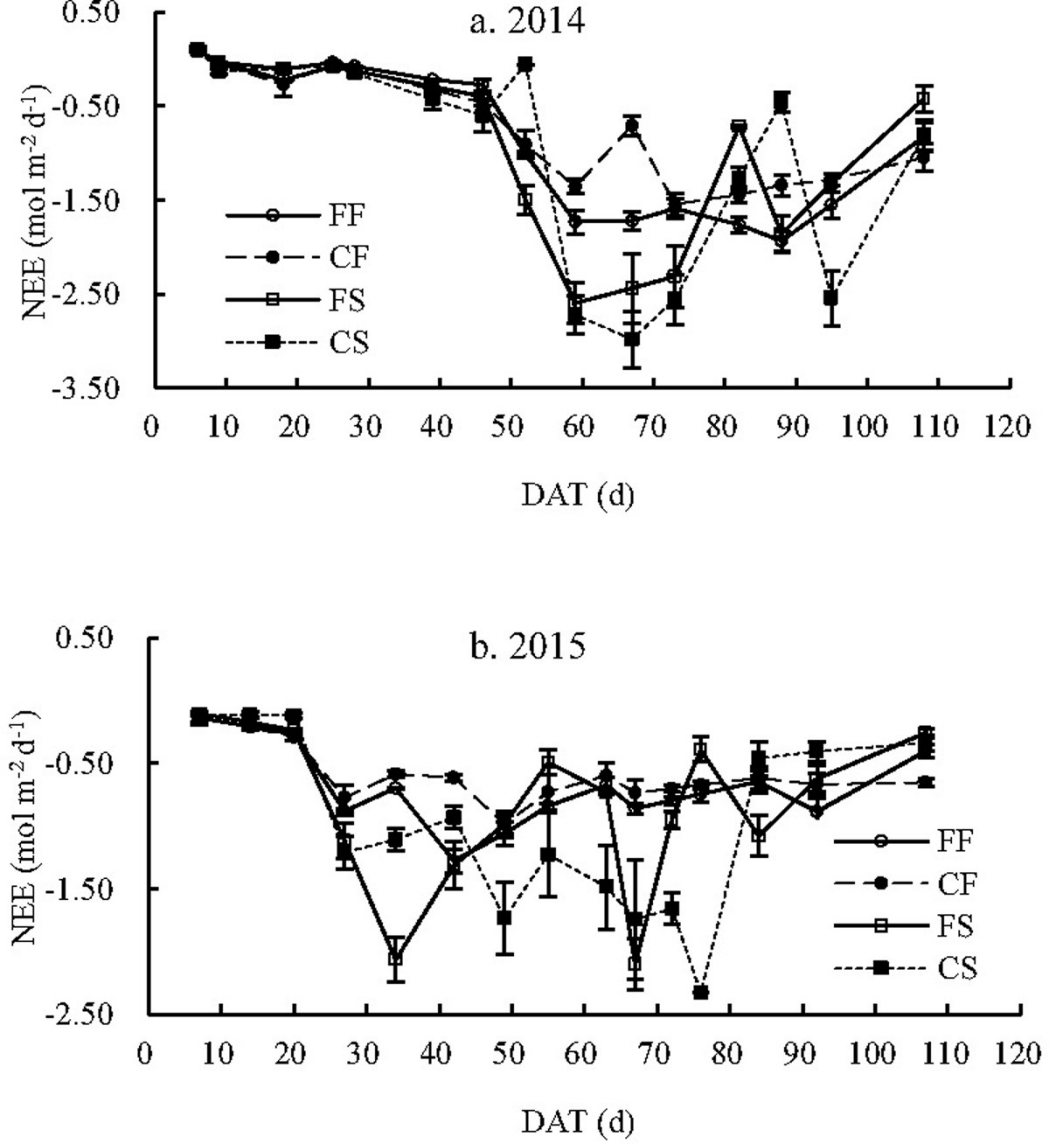
*NEE* of paddy fields with different water and carbon managements FF: Flooding irrigation and farmers’ fertilization practice, FS: Flooding irrigation and wheat straw return at a rate of 3000 kg ha^-1^, CF: Controlled irrigation and farmers’ fertilization practice, CS: Controlled irrigation and wheat straw return at a rate of 3000 kg ha^-1^, DAT: Day after transplantation.

During the early growth stage, the net CO_2_ absorption rate of the paddy field ecosystem increased with rice growth (Fig 4) and fluctuated after the peak at approximately 60–70 DAT in 2014 and 30–50 DAT in 2015. The net CO_2_ absorption rate significantly decreased with the decrease of the assimilation ability during the rice maturation stage.

The ecosystem *NEE* showed similar variations under different irrigation treatments during the early rice growth stage (before approximately 45 and 30 DAT in 2014 and 2015, respectively). Thereafter, the non-flooding periods of the WSI treatment resulted in lower net CO_2_ absorption rates of the CI paddy field ecosystems than those of the FI paddy fields under the FFP treatment until the ripening stage (107–123 DAT in 2014 and 106–121 DAT in 2015). However, the values of *NEE* for SR paddy fields under different irrigation treatments crossed each other until the ripening stage. During the ripening stage, the variation in the net CO_2_ absorption rates contrasted with the soil respiration rate. Moreover, the low net CO_2_ absorption rates for the FI paddy fields were lower than those of the CI paddy fields. For the FFP paddy fields, the average net CO_2_ absorption rates of the CF paddy fields decreased by 15.4% and 13.5% compared with those of the FF paddy fields in 2014 and 2015, respectively. For the SR paddy fields, the average net CO_2_ absorption rates of the CS paddy fields increased by 3.58% and 18.7% compared with those of the FS paddy fields in 2014 and 2015, respectively.

Overall, SR increased the net CO_2_ absorption rates of the paddy fields. However, its influence differed between different irrigation treatments. For the FI paddy fields, net CO_2_ absorption rates of the FS paddy fields were larger than those of the FF paddy fields most of the time before approximately 70 DAT. Then, the pattern reversed. For the CI paddy fields, the net CO_2_ absorption rates of the CS paddy fields were higher than those of the CF paddy fields most of time before the ripening stage. The net CO_2_ absorption rates of the CS paddy fields decreased relative to those of CF paddy fields during the ripening stage. The average net CO_2_ absorption rates of the FS paddy fields were 0.952 and 0.842 mol m^−2^ day^−1^ in 2014 and 2015, indicating an 11.58% and 21.52% increase, respectively, compared with the FF paddy fields. The average net CO_2_ absorption rates of the CS paddy fields represented a significant increase of 36.7% and 66.7% compared with the CF paddy fields in 2014 and 2015 (*p*<0.05), respectively.

Compared with traditional water and fertilizer management, the combination of WSI and SR increased the net CO_2_ absorption rates before approximately 80 DAT. Afterward, the pattern reversed, and the average net CO_2_ absorption rates increased by 15.6% and 42.2% in 2014 and 2015, respectively.

### Total *R*_*soil*_ and *NEE* throughout the rice growth stage

Table 5 shows the total CO_2_ emissions through soil respiration (total *R*_soil_) and total *NEE* of the paddy field system. Under the FFP fertilizer treatment, WSI caused an increase in total *R*_soil_ and a decrease in the total net CO_2_ absorption compared with the FI paddy fields. Total *R*_soil_ of the CF paddy fields increased by 15.2% and 8.16% compared with that of the FF paddy fields in 2014 and 2015, respectively. Total net CO_2_ absorption of the CF paddy fields decreased by 11.8% and 11.3%, respectively, compared with that of the FF paddy fields. Under the SR fertilizer treatment, WSI caused an increase in total net CO_2_ absorption (*p*<0.05) compared with FI. Total *R*_soil_ of the CS paddy fields increased by 3.88% and 3.95% compared with the FS paddy fields in 2014 and 2015, respectively. Total net CO_2_ absorption of the CS paddy fields increased by 9.73% and 13.4% compared with that of the FS paddy fields in 2014 and 2015, respectively.

**Table 5:** Total *R*_soil_ and *NEE* during the whole rice growth stage (mol m^-2^).

Year	Item	FF	FS	CF	CS
2014	*R*_soil_	52.8±1.16c	73.0±2.87a	60.8±2.62b	75.9±2.79a
	*NEE*	-115±2.37bc	-123.2±2.36b	-99.9±1.89d	-135±2.45a
2015	*R*_soil_	38.9±1.08b	105.6±3.18a	42.0±0.973b	110±2.33a
	*NEE*	-86.4±0.931c	-99.8±1.57b	-76.6±1.39d	-113±1.98a

FF: flooding irrigation and farmers’ fertilization practice, FS: flooding irrigation and wheat straw return at a rate of 3000 kg ha^-1^, CF: controlled irrigation and farmers’ fertilization practice, CS: controlled irrigation and wheat straw return at a rate of 3000 kg ha^-1^, *R*_soil_: CO_2_ emission through soil respiration, *NEE*: net CO_2_ exchange between paddy fields ecosystem and atmosphere. Means in the same line in 2014 or 2015 followed by the same letter are not significantly different (*p <* 0.05).

SR caused a significant increase in the total *R*_soil_ and net CO_2_ absorption compared with FFP management (*p*<0.05), except for the increase in total net CO_2_ absorption in 2014. The total *R*_soil_ of SR paddy fields under different irrigation treatments increased by 31.6% and 166% in 2014 and 2015, respectively, compared with that of the FF paddy fields. The total net CO_2_ absorption of SR paddy fields increased by 21.5% and 31.6% in 2014 and 2015, respectively, compared with that of the FF paddy fields.

The combination of WSI and SR resulted in a significant increase in the total *R*_soil_ and total net CO_2_ absorption compared with traditional irrigation and fertilizer treatments (*p*<0.05). Total *R*_soil_ of the CS paddy fields was 75.9 and 110 mol m^−2^ in 2014 and 2015, an increase of 43.8% and 182%, respectively, compared with that of the FF paddy fields. Total net CO_2_ absorption of the CS paddy fields was 135 and 113 mol m^−2^ in 2014 and 2015, an increase of 18.1% and 30.1%, respectively, compared with that of the FF paddy fields.

Table 6 shows the multivariate analysis of variance (MANOVA) results for total *R*_soil_ and *NEE*. The results indicated that the fertilizer treatment had a significant effect on total *R*_soil_ and *NEE*. The effect of fertilizer treatment accounted for 78.7% and 98.9% of the mean variance in the sum of squares (SS) for total *R*_soil_ in 2014 and 2015 and 68.7% and 80.0% of the total variance for total *NEE* in 2014 and 2015, respectively. The interaction of water and fertilizer treatments had a significant effect on total *NEE*. In addition, its variance contributions were 25.2% and 17.2% of the SS for the total *NEE* in 2014 and 2015, respectively. The effect of water management on total *R*_soil_ and *NEE* was not significant.

**Table 6:** MANOVA results for total *R*_soil_ and *NEE*.

Year	Influence factor	*R*_soil_			*NEE*		
		*SS*	*F*	*P*	*SS*	*F*	*P*
2014	Fertilizer management	935	51.4	9.51×10^−5^*	1.46×10^3^	93.5	1.09×10^−5^*
	Water management	88.0	4.84	5.91×10^−2^	5.54	0.355	0.568
	Interactive effect	20.0	1.10	0.325	535	34.3	3.78×10^−4^*
	Error	146			125		
2015	Fertilizer management	1.36×10^4^	1.03×10^3^	9.88×10^−10^*	1.87×10^3^	271	1.87×10^−7^*
	Water management	40.4	3.05	0.119	10.0	1.45	0.263
	Interactive effect	0.753	5.69×10^−2^	0.818	402	58.3	6.09×10^−5^*
	Error	105.9			55.2		

*R*_soil_: CO_2_ emission through soil respiration, *NEE*: net CO_2_ exchange between paddy fields ecosystem and atmosphere, *SS*: sum of squares of mean deviation.

*: significant at 0.05 level

### Soil organic carbon content

**Fig 5:**
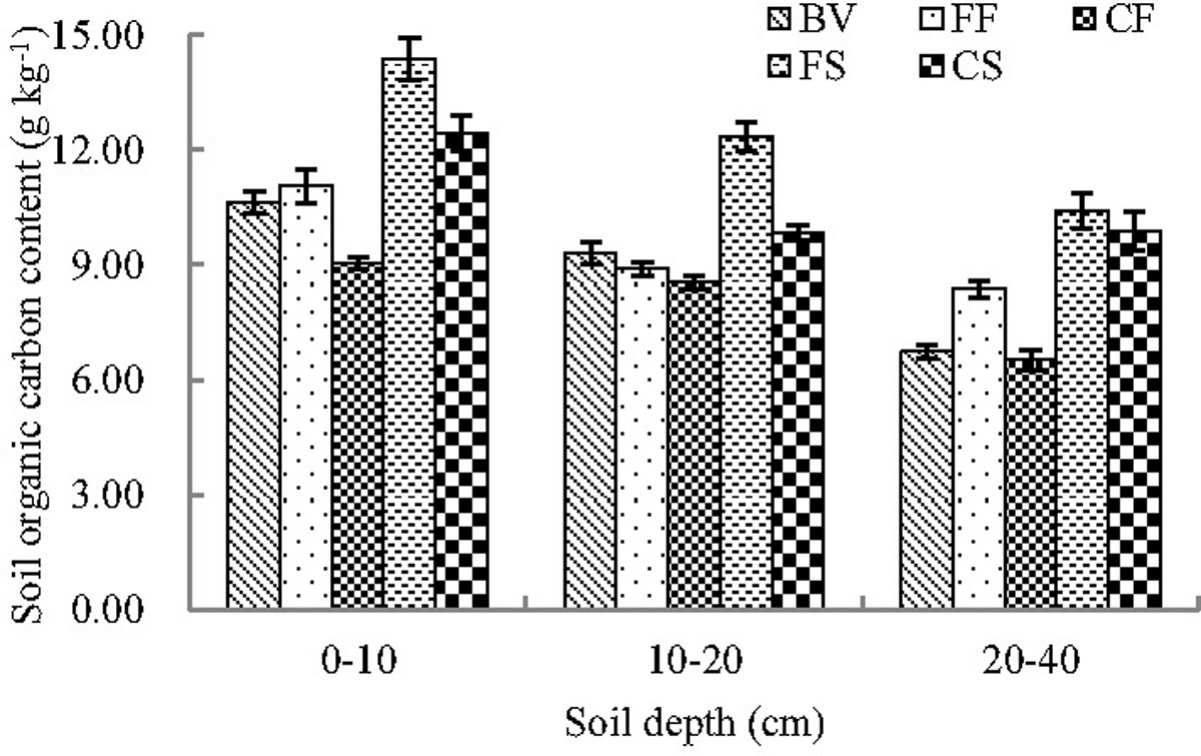
Soil organic carbon content of paddy fields before transplanting and after harvesting FF: Flooding irrigation and farmers’ fertilization practice, FS: Flooding irrigation and wheat straw return at a rate of 3000 kg ha^-1^, CF: Controlled irrigation and farmers’ fertilization practice, CS: Controlled irrigation and wheat straw return at a rate of 3000 kg ha^-1^, BV: Background value.

SR increased the postharvest soil carbon content relative to the pretransplant soil carbon content (Fig 5). Under the FS treatment, postharvest soil organic carbon content increased by 35.3%, 32.7%, and 54.8% in 0–10, 10–20, and 20–40 depths, respectively, compared with the content prior to transplanting in 2015. The rate of increase under the CS treatment was relatively low. The rates increased by 17.1%, 5.76%, and 46.7% for the three depths, respectively. SR is recommended as the most effective and economic method of soil carbon sequestration in paddy field systems [21]. SR increases carbon input, which favors fungal growth. Adhesive organic molecules associated with particulate organic matter stabilization are produced during microbial-mediated straw decomposition [22].

Pure chemical fertilizer treatment reduced postharvest soil organic carbon content relative to pretransplant content. Accelerated decomposition of soil organic carbon caused by WSI reduced soil organic carbon content after harvesting relative to the FI paddy. Xu et al. found that rain-fed fields with irrigation applied only during drought periods exhibited significantly lower total soil organic carbon stock relative to FI [23]. Non-flooding management under the CI treatment had a similar effect on soil organic carbon content to that of rain-fed management.

## Discussion

### Response of soil respiration to soil moisture and air temperature

An exponential function can used to describe the relationship between soil moisture and air temperature (Table 7). However, all the values of the coefficient of determination (*R*^*2*^) were not high. This result may be due to the influence of numerous unmeasured factors on soil respiration under field conditions. Generally, the paddy soil respiration increased exponentially with the increase in air temperature. The relationship between soil respiration and air temperature varied in different treatments. Variation in the temperature sensitivity coefficient (*Q*_*10*_) of the paddy soil respiration reveals differences between the treatments. *Q*_*10*_ values were higher under the CI treatment relative to the FI treatment as the soil respiration rate is sensitive to air temperature in the CI treatment due to the absence of an insulating water layer over the soil. Fertilizer treatment did not significantly affect *Q*_*10*_ values.

**Table 7:** The relationship between soil respiration rate and air temperature.

Treatment	Fitting equation	*R*^*2*^	*P*	*n*	*Q*_*10*_
FI	*SR* = 0.0029exp(0.1289×*T*)	0.148	<0.05	32	3.66
CI	*SR* = 0.0036exp(0.1404×*T*)	0.147	<0.05	32	4.07
FFP	*SR* = 0.0028exp(0.1373×*T*)	0.142	<0.05	32	3.95
SR	*SR* = 0.0032exp(0.1399×*T*)	0.136	<0.05	36	3.90

*SR*: soil respiration rate, *T*: air temperature, *Q*_*10*_: The temperature sensitivity coefficient of soil respiration, FI: treatments with flooding irrigation, contains FF and FS, CI: treatments with controlled irrigation, contains CF and CS, FFP: treatments with farmers’ fertilization practice, contains FF and CF, SR: treatments with wheat straw return, contains FS and CS.

**Fig 6:**
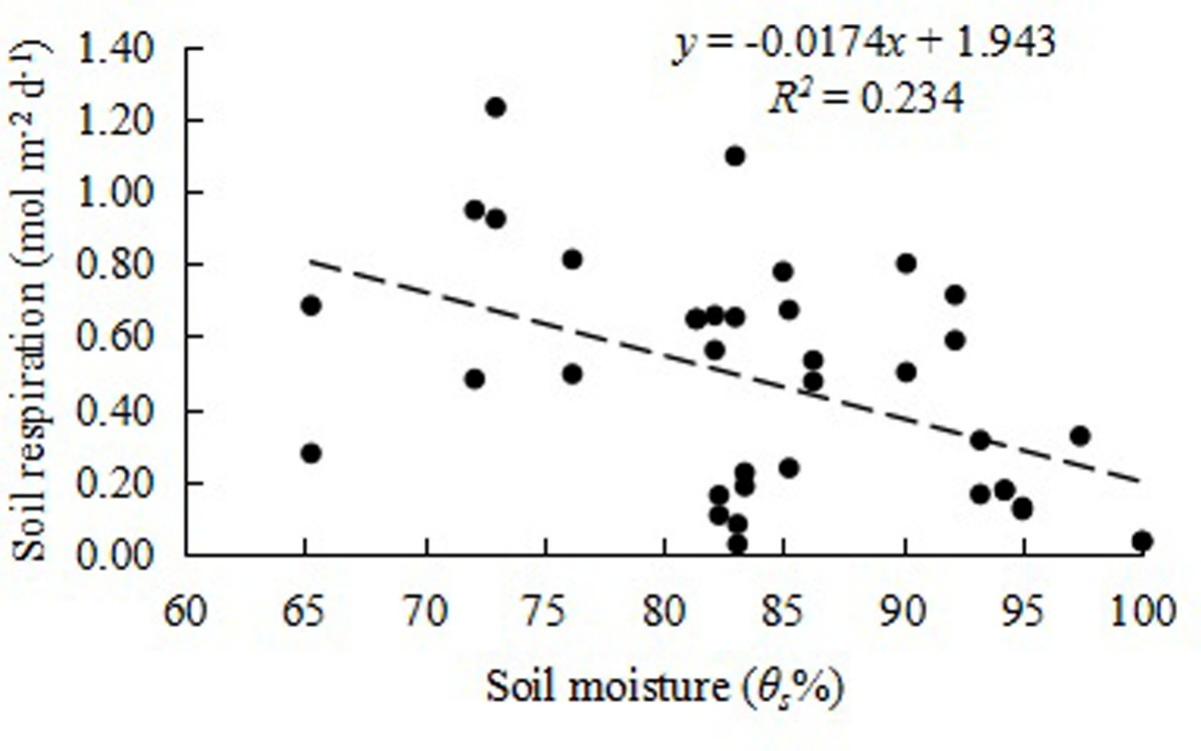
The relationship between soil respiration with controlled irrigation and soil moisture *θ*_*s*_: Volumetric soil moisture.

Soil moisture is another important factor that affects soil respiration. Soil respiration is generally believed to increase with the increase in soil moisture up to field moisture capacity. Then, the activity of aerobic microorganisms decreases under the resulting anaerobic conditions, and soil respiration decreases [24]. In this study, the water content of the WSI paddy field, which was higher than the field moisture capacity, resulted in a linear decrease in the soil respiration rate with the increase in soil water moisture (Fig 6).

### Effect of SR on soil respiration and *NEE* of the paddy fields

In this experiment, SR increased the paddy soil respiration and improved the net CO_2_ absorption of the paddy ecosystems. Most previous studies on the effects of straw addition on soil respiration and *NEE* were limited to dry farmland; this work showed that SR enhanced the soil respiration and net CO_2_ absorption in dry farmland ecosystems [25–27]. Our results showed that straw addition has the same effect on the soil respiration and *NEE* of paddy fields as in dry farmland.

The reasons for the increases in paddy soil respiration due to SR may be as follows: (1) SR increased soil porosity and CO_2_ concentration in soil solutions [28,29], and these changes facilitate the diffusion of CO_2_ from paddy soil to the atmosphere and increase CO_2_ emission. (2) SR increased soil organic matter content, thereby increasing soil CO_2_ emissions [5,30]. Existing research has shown that soil organic carbon content and soil respiration are significantly positively correlated [31,32]. In this experiment, the soil organic carbon content was higher postharvest in the SR paddy fields than that prior to transplanting (Fig 5). (3) SR increases some available soil nutrients including phosphorus, potassium, organic carbon, and alkali-hydrolyzable nitrogen [33,34]. The changes in available soil nutrients affect the rate of carbon cycling, thereby influencing soil CO_2_ emission. (4) SR affects soil microbial biomass, microbial community structure, and soil enzyme activity, promoting the metabolic activity of microbes and improving the soil respiration rate [35,36]. Meanwhile, SR promotes crop growth, yield, and dry biomass [30,37]. This increased crop growth also increased the CO_2_ absorption through rice photosynthesis. Thus, the net CO_2_ absorption of the paddy ecosystem increases along with the increased soil respiration rates in paddy fields under SR management relative to paddy fields without SR.

The increased respiration and net CO_2_ absorption of paddy soil under SR exhibited significant interannual variation in this experiment (Table 5). The effects of SR in the second year (2015) were greater than that in the first year (2014), particularly for paddy soil respiration values. This phenomenon can be attributed to the slow decomposition rate of straw [38,39]. The reductive conditions of paddy fields were also an obstacle to straw decomposition compared with that of dry farmlands. This restricted the effect of SR on promoting paddy soil respiration and net CO_2_ absorption for the first year. Dry farmland management during the rain-fed wheat stage (all the plots had the same field management during the rain-fed wheat stage) accelerated wheat straw decomposition and supplied reaction substrate for soil microorganisms and animals as well as nutrients for rice growth during the next season. This process resulted in the increased effect of straw on paddy soil respiration and net CO_2_ absorption, which became more apparent in the second year. In addition, continuous application of wheat straw for two years may also help to explain this interannual variation. Previous research has shown that cotton soil respiration increases with the increase in SR over years of continuous cropping, and over 30 years of continuous SR increases cumulative CO_2_ emission through soil respiration by 4.26% compared with that in the fifth year [40].

### Effects of water and straw management on rice production and CO_2_ exchange of paddy fields

A previous study has shown that various WSI management modes in China can reduce the volume of irrigation by 8%–50% and increase rice yield and irrigation water use efficiency by 3.00%–8.00% and 20.0%–80.0%, respectively [41]. A meta-analysis showed that alternate wetting and drying (AWD) decreased rice yields by 5.40% and irrigation water input by 25.7% compared with continuous flooding in Asia; however, under mild AWD, rice yields were not significantly reduced in most circumstances. As a result, AWD increased irrigation water use efficiency by 24.2% [42]. WSI caused a slight decrease in rice yield in this study. In addition, WSI decreased the net CO_2_ absorption of a paddy field ecosystem and soil organic carbon content by 9.73%–13.4% and 3.24%–20.3%, respectively, compared with FI (Table 5 and Fig 5). As a farmland management technology widely recommended in China, SR can decrease chemical inputs, promote soil C sequestration, and improve crop yields [3,4]. This research determined whether SR can resolve decreased rice yield and decreased net ecosystem CO_2_ absorption of paddy fields under WSI. The results of this study showed that the joint regulation of WSI and SR reduced the irrigation water input by 43.3%–49.5% and increased rice yield by 3.77%–7.14% compared with traditional water and fertilizer management practices. Meanwhile, total net CO_2_ absorption and soil organic carbon content increased by 18.1%–30.1% and 5.76%–46.7%, respectively. Therefore, the joint regulation of WSI and SR is an effective measure for maintaining yield, increasing irrigation water use efficiency, mitigating CO_2_ emission, and promoting paddy soil fertility. In addition to CO_2_, paddy fields are an important emission source of CH_4_ and N_2_O. Generally, SR markedly increases CH_4_ and N_2_O emissions from paddy fields under FI [4,43]. WSI can reduce CH_4_ emission and increase N_2_O emission from paddy fields [44,45]. However, few studies have focused on the effect of SR on CH_4_ and N_2_O emissions from paddy fields under WSI. WSI and SR techniques have been widely applied in paddy fields in China. Therefore, the greenhouse effects (CH_4_, N_2_O, and CO_2_) of paddy fields with joint regulation of SR and WSI should be studied in depth.

The excessive nitrogen fertilizer input into paddy fields in China is also an urgent issue. Nitrogen fertilizer input during the rice season in the Taihu Lake region, which is one of the main rice-producing areas of China, reaches up to 270–300 kg N ha^−1^ [46]. In this experiment, nitrogen fertilizer inputs, according to local conventional fertilizer application during the rice season, were 291 and 283 kg N ha^−1^ in 2014 and 2015, respectively. However, the recommended amount of nitrogen fertilizer input is only 190–200 kg N ha^−1^ [47]. In addition, the nitrogen use efficiency of paddy fields was only approximately 30.0% [48]. Much of the applied nitrogen fertilizer is lost through runoff, leaching, and ammonia volatilization. High rates of nitrogen fertilizer input and the loss of paddy fields in this region are contributors to the eutrophication of lakes and rivers. Therefore, measures such as introducing N fertilizer tax, improving local extension services, and educating farmers for environmental awareness should be taken to avoid excessive nitrogen fertilizer input and serious environmental degradation in the Taihu Lake region.

## Conclusions

Understanding the effects of WSI combined with SR on the CO_2_ exchange between a paddy field ecosystem and the atmosphere can support comprehensive evaluations of greenhouse effects and the sustainable use of the water and carbon resources of paddy fields. CS management significantly increased the rice yields and the irrigation water use efficiency of paddy fields compared with the control. CS clearly increased soil respiration rates during most of the rice growing season and increased net CO_2_ absorption rates before approximately 80 DAT. Afterward, the pattern reversed. Total CO_2_ emission through soil respiration of CS paddy fields increased by 43.7% and 182% compared with the control in 2014 and 2015, respectively. However, CS also raised the total net CO_2_ absorption by 18.1% and 30.1% in these two years, respectively. Frequent alternating wet–dry cycles of the CI paddy fields led to an increase in soil respiration and a decrease in net CO_2_ absorption. SR promoted paddy soil respiration but also increased the net CO_2_ absorption and paddy soil organic carbon content. The present study concludes that the joint regulation of WSI and SR is an effective measure for maintaining yield, increasing irrigation water use efficiency, mitigating CO_2_ emission, and promoting paddy soil fertility.

## Supporting information

S1 Data File We provided the data used in this research. The caption “Fig 2” mentioned at Fig 2 as an example.
(XLSX)
